# Automated Room-Level Localisation Using Building Plan Information

**DOI:** 10.3390/s24175753

**Published:** 2024-09-04

**Authors:** Mathias Thorsager, Sune Kroeyer, Adham Taha, Magnus Melgaard, Linette Anil, Jimmy Nielsen, Tatiana Madsen

**Affiliations:** Department of Electronic Systems, Aalborg University, 9220 Aalborg, Denmarkjjn@es.aau.dk (J.N.); tatiana@es.aau.dk (T.M.)

**Keywords:** indoor localisation, RSSI fingerprinting, building management system

## Abstract

Building Management Systems (BMSs) are transitioning from utilising wired installations to wireless Internet of Things (IoT) sensors and actuators. This shift introduces the requirement of robust localisation methods which can link the installed sensors to the correct Control Units (CTUs) which will facilitate continued communication. In order to lessen the installation burden on the technicians, the installation process should be made more complicated by the localisation method. We propose an automated version of the fingerprinting-based localisation method which estimates the location of sensors with room-level accuracy. This approach can be used for initialisation and maintenance of BMSs without introducing additional manual labour from the technician installing the sensors. The method is extended to two proposed localisation methods which take advantage of knowledge present in the building plan regarding the distribution of sensors in each room to estimate the location of groups of sensors at the same time. Through tests using a simulation environment based on a Bluetooth-based measurement campaign, the proposed methods showed an improved accuracy from the baseline automated fingerprinting method. The results showed an error rate of 1 in 20 sensors (if the number of sensors per room is known) or as few as 1 per 200 sensors (if a group of sensors are deployed and detected together for one room at a time).

## 1. Introduction

In Building Management Systems (BMSs) for monitoring and controlling the quality of the indoor environment, the used sensors and actuators are often deployed in fixed positions using wires for power and network connections. Recently, however, the Internet of Things (IoT) wave has made wireless sensors and actuators attractive for BMSs. Such wireless solutions are less costly to deploy (since fewer cables are needed and the installation can be conducted more quickly) and allow for flexibility in room configurations as sensors can be easily repositioned. However, as the wires connecting the sensors to the Control Units (CTUs) are removed, ensuring that all sensors and actuators communicate with the correct CTUs becomes a challenge. If the BMS does not know which room a sensor is in, the sensor cannot communicate with the correct CTU, which means that Heating, Ventilation, and Air Conditioning (HVAC) functions cannot work effectively and efficiently, and while traditional wired systems rely on this mapping being correctly configured by a technician upon installation of the BMS, a flexible wireless system should ideally discover in which room it is located and notify the BMS accordingly. This means that the BMS system should be able to discover the room locations of the sensors and use this information to map them to the correct CTU without requiring extensive manual work by the technicians—otherwise, the benefit of using a wireless solution would be lost. This requires the use of a wireless localisation method which can accurately estimate which room a sensor is in.

**Figure 1:**
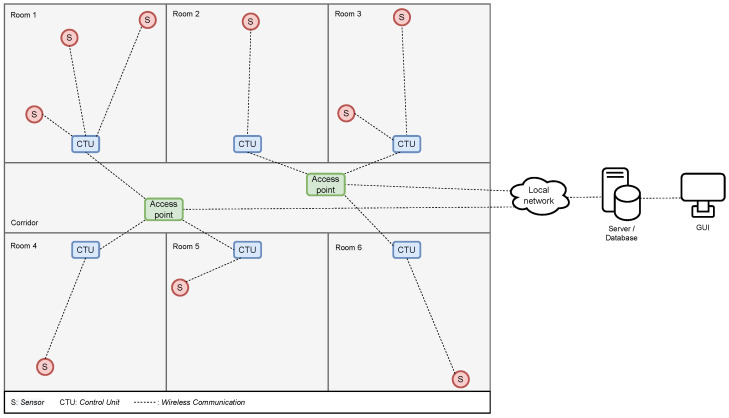
General structure of the communication between the BMS hardware devices in a building plan consisting of six rooms. The red circles indicate IoT devices such as sensors, the blue boxes are control units which connect to the sensors and receive the sensing data, and the green boxes are access points which forward data from CTUs to the BMS.

Figure 1 shows a typical scenario where sensors spread out over several rooms in a building must be connected to appropriate CTUs. In the scenario, the CTUs can communicate directly with each other through a wireless link. The CTUs can also communicate wirelessly with the sensors in range, which can be all or a subset of the sensors, depending on the size of the building. Furthermore, the CTUs can relay information gathered from the sensors to a centralised server via the building’s WiFi access points. The CTU-to-CTU and CTU-to-sensor links can either be maintained through the access points or as direct device-to-device links using technologies such as Bluetooth, Zigbee, Ultra-Wideband (UWB), etc., depending on CTUs and sensors hardware capabilities.

The CTUs additionally function as Graphical User Interfaces (GUIs), from which the indoor climate and actuators can be controlled by people in the rooms, and are therefore assumed to be mounted on the wall next to the door inside each room. Furthermore, it is assumed that the mounting position is known for all CTUs through information from a building plan in the BMS.

Similarly, this building plan likely contains information that can be used to improve the localisation accuracy of localisation methods. This information could include the number of sensors in each room, the signal attenuation based on the materials of the walls in the building, etc. Whether the information from the building plan can be used to enhance the accuracy of the estimated locations will depend on the type of localisation method used. For instance, if a multilateration-based localisation method is used in an indoor environment, knowing the attenuation of walls can help to more accurately estimate the expected path loss for positions with non-line of sight signal paths. Similarly, by knowing how many sensors are in each room, a fingerprinting approach can add additional restrictions on the estimations of the individual sensors by taking into consideration prior and possibly future estimations of sensors in the building.

In this paper, we propose three room-level localisation methods, which are divided into two main categories: (1) An automated fingerprinting-based single-sensor method, which estimates the location of a single sensor at a time. (2) Two multi-sensor methods, which rely on imposing constraints that are typically reasonable, namely (a) that the number of devices installed in each room is known, or (b) that all devices to locate are in the same room (e.g., after installing sensors in a room, the technician triggers an automatic room-detection process). Both multi-sensor methods use this information to estimate the location of a group of sensors at once, and while fingerprinting-based localisation methods are not new to the literature, the current state-of-the-art (SoA)focuses on improving the distance-based accuracy of the localisation methods. Different from this, the proposed methods solve the problem of enabling indoor localisation of static off-the-shelf sensor devices in scenarios where no extensive manual work (e.g., creating RSSI maps) can be afforded. To the best of the authors’ knowledge, this is a scenario which has not been studied in the literature.

For evaluating the considered localisation algorithms, a measurement campaign was conducted at the dept. of Electronic Systems, Aalborg University to characterise the radio environment experienced by off-the-shelf Bluetooth-based IoT devices. The measurements were obtained in indoor environments for both Line of Sight (LoS) and Non LoS (LoS) situations, with the goals of (1) understanding the impact of distance and walls between receiver and transmitter devices, and (2) choosing and parameterising a suitable path-loss model to be able to realistically simulate the environment. The considered algorithms are evaluated both through extensive simulations and in real-life validation scenarios.

We show that when considering room-level localisation, the single-sensor method maintains a comparable performance to an unrestricted fingerprinting approach with a dense grid of Anchor Points (APs) in each room. Additionally, the proposed multi-sensor methods provide significant improvements to the localisation accuracy of the automated single-sensor method. They reach a slightly better performance than the current SoA fingerprinting-based localisation methods of room-level accuracy.

This paper is structured as follows: Section 2 presents the SoA for localisation methods, Section 3 briefly presents the system model following the room-mapping concept, after which it introduces and defines the proposed localisation methods. Section 4 describes the conducted measurement campaign and derived environment models, whereas Section 5 presents the performance evaluation of the proposed methods, and finally, Section 6 concludes this paper.

## 2. Related Work

A large body of literature exists, studying the general problem of determining the geographic location of a wireless device from radio measurements. This problem is generally referred to as localisation. Localisation methods are based on three aspects: the technology (WiFi, Bluetooth, 4/5G, Ultra-Wide Band (UWB) etc.), the measurement technique (Received Signal Strength Indicator (RSSI), Channel State Information (CSI), Angle of Arrival (AoA), Time Difference of Arrival (TDoA), etc.), and the localisation technique (multiangulation, multilateration, fingerprinting, etc.).

In recent years, UWB has seen a surge in popularity due to its high localisation accuracy, which is largely unrivalled by other technologies. In indoor localisation, localisation methods utilising UWB’s precise time of flight ranging (the calculation of distance between transmitter and receiver) reach localisation accuracies up to 10 cm [1]. However, despite its high accuracy in indoor localisation, UWB requires specialised hardware, which remains a high-cost solution, and is mainly recommended for use in industrial IoT where localisation accuracy is critical [2]. Similar to UWB, Visible Light localisation (VLL) has also shown great performance in localisation accuracy achieving estimation errors in the mm range [3]. VLL utilises a wide range of techniques to perform localisation ranging from classical distance- or AoA-based localisation [4] to spatial division-based approaches such as spatial beam localisation [5] and spatial encoded projection [6]. The main drawbacks of VLL is that there is a strict requirement of LoS between the transmitter and receiver. Where Radio Frequency (RF) transmissions can penetrate objects at the cost of some signal strength, visible light cannot (unless the object is transparent). Even though there may be a receiver, in the form of a CTU, in each room, obstacles such as tables, chairs, etc. will significantly impact the accuracy of the localisation and in some cases make it impossible to locate a sensor. Furthermore, while the hardware needed for VLL is not expensive, it is not guaranteed that all sensors nor the CTUs used in an office building will be equipped to handle VLL.

In outdoor localisation, multilateration or -angulation are widely used localisation techniques [7,8]. However, due to the obstruction of walls, these techniques are more inaccurate when used in indoor localisation. Multilateration requires the conversion of measurement (RSSI, Time of Arrival (ToA)) to a distance which is greatly impacted by the multipath effect caused by walls and other objects [1], and while multiangulation is also affected by multipath, there are methods for estimating the AoA in noisy environments [8]. As such, the biggest hurdle for using AoA as the measurement technique is the requirement of specialised hardware, which defeats the purpose of the cost savings introduced by the wireless BMS.

In the current literature, the most prevalent indoor localisation technique is the fingerprinting method using RSSI measurements [9]. The fingerprinting localisation technique consists of two phases: (1) An offline phase where the environment is divided into grid points known as APs, and for each point a so-called fingerprint of, e.g., RSSI levels to in-range access points, which in the case of a BMS would be the CTUs, are stored in a database; (2) an online phase where the measurements of a sensor towards in-range CTUs is compared against the database of fingerprints to determine the most likely location of the sensor. Fingerprinting using RSSI requires no specialised hardware and has been shown to achieve sub-meter accuracy [10]. The accuracy is often attributed to the density of the APs with a higher density allowing for higher precision in location estimates. However, the offline phase of populating the fingerprint database is typically a laborious task. As such, advances in fingerprinting-based localisation techniques have focused on improving the accuracy of the estimates without simply increasing the density of the APs [11]. Depending on the scenario of the localisation, this is conducted in different ways. For moving targets, filters such as Kalman filters [12] and Bayesian filters [13] are used to increase the accuracy of estimates over time. For stationary targets, and as an addition to filters with moving targets, interpolation methods are used to reach a continuous estimation space rather than a discrete space [14,15,16,17].

Considering the particular problem of interest for a BMS, i.e., determining just the room in which a wireless sensor is located, a localisation method used for a BMS does not require SoA distance precision. Instead, what is important is that the localisation method is able to consistently determine the location of a sensor with room-level precision without introducing additional manual work for the technician who is installing the sensors; while the problem of room-level localisation is seemingly less strict than traditional localisation, this problem has only been sparingly considered in the literature. In most of the literature, the room-level localisation schemes utilise RSSI-based fingerprinting; however, they utilise several anchor nodes in each room [18,19]. Alternatively, some authors present a different approach to the room-level localisation [20] where devices are detected when they cross a boundary point (door frame). In order to utilise such localisation methods, a separate installation of the localisation sensors must be performed before the installation of the sensors, which should be located. As such, this approach is deemed impractical in a BMS installation setting.

## 3. Proposed Methods

The localisation schemes presented in this paper use an automated RSSI-based fingerprinting method. The fingerprinting scheme is chosen as it is among the most commonly used localisation methods for indoor localisation while still remaining a simple and flexible method [9]. These are two important characteristics for use with BMSs as it is not always possible to control the specific hardware which will be used for the localisation due to frequent cost-saving measures. A relevant localisation method must therefore be able to be retro-fitted to existing hardware and still work well in indoor propagation environments. Furthermore, as the literature has shown when considering room-level localisation accuracy, fingerprinting-based methods can achieve great performance.

In order to use fingerprinting, the reception of a transmission at multiple receivers is necessary. In Bluetooth, devices can communicate with multiple other devices through either multiple active connections or by utilising the advertisement transmissions. Furthermore, as Bluetooth is one of the most commonly used technologies in wireless sensor networks [21], the localisation methods proposed in this paper are based on transmissions made over Bluetooth protocol. This means that for the localisation, the sensors transmit Bluetooth messages to all CTUs which in turn measure the RSSI of the received messages.

Fingerprinting can be defined and utilised in many ways, but for this paper, fingerprinting is defined as follows. A fingerprint is a set of RSSI measurements recorded at each in-range CTU from a transmission at a single known location–the AP. This set of measurements now acts as a fingerprint for that specific AP. As such, fingerprinting is the act of collecting several fingerprints of different APs in a building. This set of fingerprints can be used to estimate the location of a sensor by comparing the sensor’s fingerprint with the set of pre-measured fingerprints. The comparison is commonly conducted using Mean Squared Error (MSE), where the estimated location is the AP with the best matching fingerprint, the AP whose fingerprint has the lowest MSE, as follows:

(1) $$\begin{array}{c} F_{*}=\underset{F_{m}\in F}{\mathrm{argmin}}\frac{1}{N}\sum_{n=1}^{N}{\left(W_{m,n}-V_{n}\right)}^{2}, \end{array}$$

where $F_{*}$ is the fingerprint which is the best match to the sensor, $F=\{F_{1},F_{2},\cdots ,F_{M}\}$ is the set of all pre-measured fingerprints, $V_{n}$ is the *n*th RSSI value of the fingerprint from the sensor whose position is being estimated, and $W_{m,n}$ is the *n*th RSSI value of the fingerprint from CTU *m*.

In case there are values missing in a fingerprint, for example, if the attenuation between a sensor and CTU is too great for the CTU to detect the signal, a default value equal to the threshold for detection of −100 dBm is used in fingerprint comparison. The CTU cannot send this value itself, as it is not aware that a fingerprint transmission has been sent; however, as the RSSI values are sent to the server shown in Figure 1, the server will know which CTUs did not send a value and record the default value in their stead. While the specifications of Bluetooth require a device to be able to detect signals at −82 dBm, actual implementations hit a target of −100 dBm instead [22]. Furthermore, we assume that the CTUs are not equipped with a full duplex transceiver, and as such, they cannot measure the RSSI of their own signal. Instead, they simply record an RSSI value of 0 dBm. The value of 0 dbm itself is not necessarily important. What is important is that a value is recorded such that a complete fingerprint is created. The rationale behind the choice of specifically 0 dbm is based on the desired outcome of the fingerprinting localisation. When a sensor transmits from a room, the CTU, which will record the highest RSSI value, is most often the CTU, which is in the same room as the sensor. As such, the fingerprint of the CTU in that room should have the highest saved RSSI value of all the CTUs. Since a Bluetooth sensor will likely transmit at or below 0 dbm [22], 0 dbm was chosen to represent a transmission of 0 m.

### 3.1. Single-Sensor Method-Automated Fingerprinting

The common practice when using fingerprinting-based methods is to divide the area where the sensors can be placed into a grid and use each grid location as an AP. Using MSE as the final step in fingerprinting to find the location of a sensor is a rather simple approach compared to the SoA of fingerprinting methods. With MSE, we cannot interpolate between APs. As such, the accuracy of the location estimates, in terms of distance from the true location, is mainly dependent on the resolution of the grid. However, as the goal is to estimate the room in which the sensor is located, rather than achieve an accurate estimation of the sensor’s precise location, the MSE approach suffices.

Furthermore, since measuring a dense grid of APs requires significant manual work, as the fingerprint of each AP must be measured manually, performing this extensive offline fingerprinting would go against the requirement of automating the setup process. Our proposed automated fingerprinting method avoids this problem by removing the majority of the APs. Instead of using multiple APs in each room, we limit the amount to one AP per room. Since the CTUs can communicate directly with each other, the locations of these are obvious candidates for the APs. This means that the offline phase for our proposed fingerprinting can be initiated automatically when the CTUs are installed in each room, and it can be repeated periodically to keep the fingerprints up to date in case the environment changes in the rooms.

### 3.2. Multi-Sensor Methods

Instead of locating sensors individually, we can use information present in the building plan regarding the distribution of sensors in each room. Not only are we including more information in the localisation process, but estimating multiple sensors at once will also help negate some of the individual outlier estimation errors that can occur for single-sensor estimates. We propose two methods for estimating the locations of a group of sensors. Each of the methods requires the initial use of a single-sensor localisation method. The two multi-sensor methods are therefore not presented as standalone localisation methods but as extensions to automated fingerprinting.

#### 3.2.1. Most Probable Permutation

The first of the multi-sensor methods, Most Probable Permutation (MPP), requires that the number of sensors in each room is known. We argue that this information is available from the building plan, since a technician will be installing the sensors based on the specifications of the building plan. The information is strictly used to constrain the amount of sensors that are estimated in a room by the MPP localisation method. With this information, the goal is to find the permutation of sensors placed in the different rooms, which minimises an error function. For this, we introduce two vectors, *Y* and *Z*, which contain the sensor IDs and references to the room in which each sensor is placed, respectively. Take, for example, a building with four rooms (A, B, C, D) and six sensors with IDs Y=[1,2,3,4,5,6]. Given a distribution of (2,0,1,3)—i.e., room A has two sensors, room B has 0 sensors, room C has 1 sensor, and room D has 3 sensors—a possible permutation is Z=[A,C,A,D,D,D], which means that sensors 1 and 3 are placed in room A, sensor 2 is in room C, and sensors 4, 5, and 6 are in room D. With two such vectors, *Y* and *Z*, the error is calculated using the summed MSE E of each individual sensor, as follows:

(2) $$\begin{array}{c} E\left(Y,Z\right)=\sum_{i=1}^{\left|Y\right|}\left(\frac{1}{N}\sum_{n=1}^{N}{\left(W_{Z_{i},n}-V_{Y_{i},n}\right)}^{2}\right), \end{array}$$

where $W_{Z_{i},n}$ is the *n*th RSSI value of the CTU in the room specified by $Z_{i}$, and $V_{Y_{i},n}$ is the *n*th RSSI value of the sensor with ID $Y_{i}$.

Based on (2), the best permutation $Z^{*}$ is found as the permutation which minimises E(Y,Z):

(3) $$\begin{array}{c} Z^{*}=\underset{Z}{\mathrm{argmin}}E\left(Y,Z\right), \end{array}$$

A weakness of the MPP method is that it requires correct information on the number of installed sensors in a room. In the following, we present another method with more relaxed requirements.

#### 3.2.2. All Sensors One Room

The second multi-sensor method covers a simpler case, where all sensors in a group are known to be in the same room, hence the name All Sensors One Room (ASOR). While we know that all sensors are in the same room, we do not know which room they are in. As such, the goal of the ASOR method is to determine which room has the highest likelihood of containing all the sensors. Unlike the MPP method, the ASOR method is not based on information from the building plan. Instead, it is based on the technician initiating the localisation process for all sensors in a single room at the same time, one room at a time. Given again six sensors Y=[1,2⋯,6], the location of each sensor is estimated individually using (1). The result is a vector with the same length as *Y*, which contains the rooms that each sensor is estimated to be in. The final estimate for all sensors is then the room in which most of the sensors were estimated to be. As long as the majority of sensors are estimated in the correct room, all sensors in the group are estimated correctly. However, this also means that if only a minority of the sensors are estimated correctly, none of the sensors are estimated correctly in the end.

## 4. Evaluation

For testing the proposed methods, we developed a simulator that can generate realistic RSSI values for a given building environment, accounting for distance-related path loss and wall attenuation between transmitter and receiver, as well as small-scale fading. The model was fitted to data from a measurement campaign conducted at the dept. of Electronic Systems, Aalborg University. The RSSI model is a modified log-normal path loss model [23] that takes into account the signal attenuation caused by walls between the transmitter and receiver [24], as follows:

(4) $$\begin{array}{c} w\left(d\right)=-P_{0}-10\alpha \log_{10}\left(d\right)-\beta \cdot m-\xi , \end{array}$$

where *d* is the distance in meters between the transmitter and receiver, $P_{0}$ is the reference power at 1 m distance for a fixed transmit power, α is the path loss exponent, β is the Wall Attenuation Factor (WAF) in dB, *m* is the number of walls between the transmitter and receiver, and ξ is the small-scale fading which follows a zero mean Gaussian distribution [23]. Variables $P_{0}$, α, and β are all fitted to the data obtained during the measurement campaign.

### Measurement Campaign

**Figure 2:**
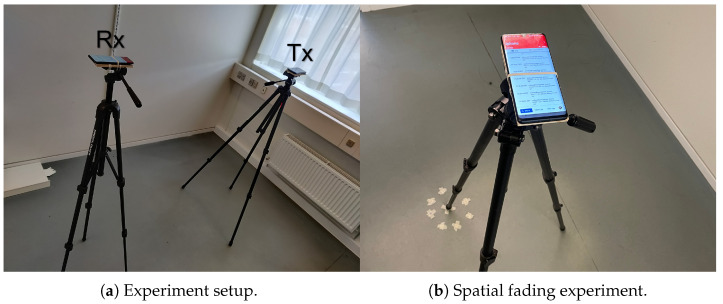
Experimental setup of RSSI measurements using two mobile phones. (**a**) Shows the equipment used for all measurements, and (**b**) shows the additional measurement locations for the transmitter to counteract the spatial fading.

**Figure 3:**
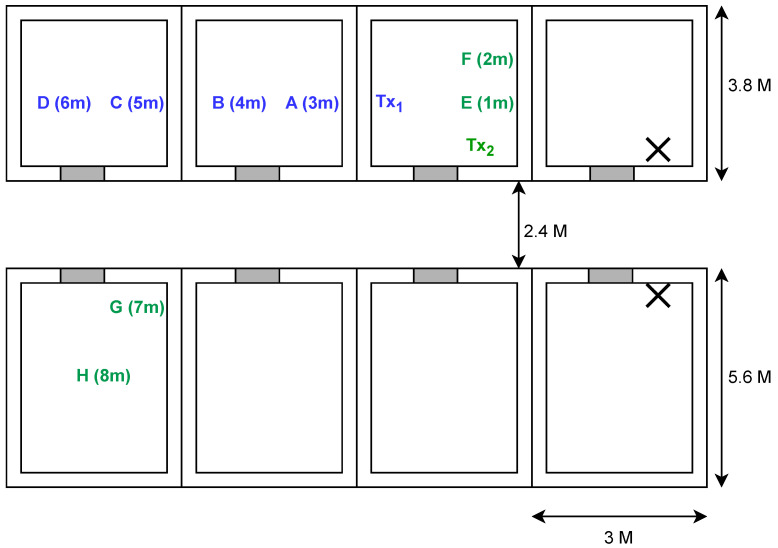
Building plan used for the measurement campaign as well as the tests. Tx_1_ and Tx_2_ depict the transmitter locations for the measurement campaign with the coloured letters depicting the receiver locations for the respective transmitter locations. The two Xs indicate the locations of the CTUs repeated in each room.

The measurement campaign was conducted using two mobile phones placed on camera tripods, as shown in Figure 2a, as these devices are assumed to be representative of the sensors and CTUs used in a real BMS, and while more accurate measuring devices would be able to remove more of the noise from the hardware, we argue that if we can capture and model the noise in the RSSI model, we will end up with a simulator that more closely represents the use case. By carrying out the measurements in empty offices, we ensured that the environment would remain static throughout the campaign, and while the empty offices do impact the variables of the RSSI model (in particular the path loss exponent) we overcome this in the testing of the methods by including tests that vary the parameters of the model individually to simulate differences in the environment. The measurements were carried out by placing the transmitter in a static position and moving the receiver to different positions of increasing distance. The location of the receiver follows the marked locations on Figure 3. These locations provide measurements of different distances between the transmitter and receiver as well as providing measurements in LoS and NLoS conditions. By using such measurements the model will likewise be able to generate measurements for LoS as well as NLoS transmissions by simply specifying the amount of walls between the transmitter and receiver.

When measuring at specified locations, there are two sources of randomness which we took care to mitigate. These are the temporal variations in RSSI, which can be observed as variations in RSSI for consecutive transmission between a stationary transmitter and receiver, and a spatially dependent small-scale fading which occurs when either of the devices is moved (even slightly) or changes in the environment leads to a different multi-path fading. The temporal variations are mitigated by measuring several consecutive transmissions from the exact same location and taking the average RSSI value. The spatial fading is mitigated in a similar fashion by measuring at several locations at a distance corresponding to half a wavelength of the original position. It is further assumed that the temporal noise can be mitigated during the real use of the localisation methods since it would simply require the sensors to transmit multiple times without moving them. The spatial fading is not as simple. Since it requires moving the receiver (or transmitter) to mitigate the fading, this would go against the goal of the localisation method being automated. Thus, we include the random variable ξ to represent the spatial fading uncertainty.

The procedure for measuring at each location is as follows:

1. Place the transmitter and receiver in the locations specified in Figure 3.
2. For each of the markings *i* at location *l* as indicated by Figure 2b, measure N=200 consecutive RSSI values and compute the sample mean as ${\hat{\mu }}_{i}^{l}=\frac{1}{N}\sum_{n=0}^{N}{w^{\prime }}_{i}^{l}\left(n\right)$, where ${w^{\prime }}_{i}^{l}\left(n\right)$ is the *n*th RSSI measurement for the *i*th marking at location *l*.
3. Calculate sample mean ${\hat{\mu }}_{\xi }^{l}=\frac{1}{I}\sum_{i=0}^{I}{\hat{\mu }}_{i}^{l}$ and sample standard deviation ${\hat{\sigma }}_{\xi }^{l}=\sqrt{\frac{{(\sum_{i=0}^{I}{\hat{\mu }}_{i}^{l}-{\hat{\mu }}_{\xi }^{l})}^{2}}{I}}$ of the spatial fading random variable ξ for each location *l*.

**Figure 4:**
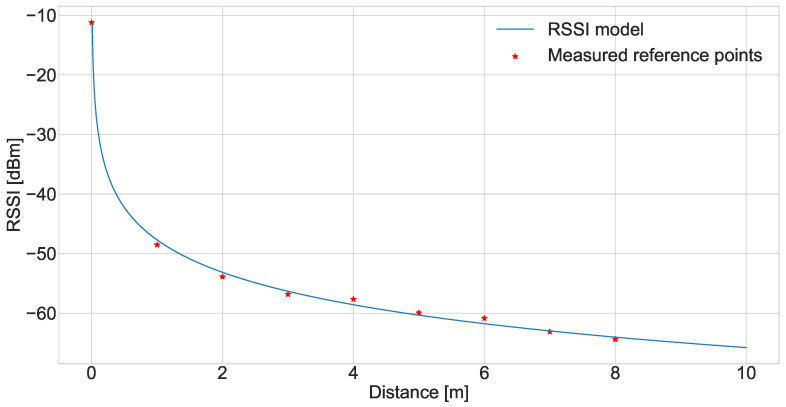
Fitted RSSI model using parameters shown in Table 1 plotted against the measured values shown in Figure 3 normalised for the wall attenuation. The point close to 0 m is an additional measurement point taken with both measurement devices placed in the same location.

The parameters, $P_{0}$, α, and β, of the RSSI model are found through maximisation of the coefficient of determination $R^{2}$ when fitting the RSSI model to the data points. In order to do this, the distance and number of walls between the transmitter and receiver are linked to each RSSI measurement. The distance is taken based on the direct path between the transmitter and receiver and the number of walls is counted based on them intersecting with this same path. The parameters which resulted in the largest $R^{2}$ value are shown in Table 1. These values resulted in an $R^{2}$ value of 0.9986, which indicates a strong fit with the measured data, as also shown in Figure 4. The values of both the path loss exponent and material penetration attenuation are well studied in the 2.4 GHz domain. By comparing the resulting parameter values from the measurement campaign with the expected values from the literature, we see that the path loss exponent is on the lower end of the scale, where a path loss exponent of 1.8 typically indicates a free space path loss environment [25]. Considering that we have isolated the wall attenuation contribution in a separate term, our path loss exponent in fact only represents the free space contribution to the total path loss. For the WAF, the measured value of 2.3 dB is within the expected range given the material of the walls in the measurement location [26].

## 5. Results

This section evaluates the performance of the proposed localisation methods based on extensive Monte Carlo simulations. Due to the restrictions presented by the requirement of automated localisation, the current SoA localisation methods are not applicable. However, for transparency purposes, we include two well-known localisation methods to compare against our proposed automated fingerprinting-based localisation scheme. In particular, we compare against (1) fingerprinting methods which employ a grid of APs in each room and (2) the multilateration method. The purpose of including the traditional fingerprinting method is to show the loss in accuracy when only a single AP is used per room. This is shown using both a simple extension of the MSE-based fingerprinting used in this paper, referred to as the extended fingerprinting method in the following sections and a selection of SoA room-level accuracy fingerprinting-based localisation methods. While the multilateration method is known to perform poorly in indoor localisation due to the effects of the severe multipath propagation [1], to the best of our knowledge, it is the only other automated localisation method which can use RSSI measurements (if the hardware allowed for AoA estimates, the multiangulation method could have also been used; however, this similarly suffers from the effects of indoor multipath propagation). The multilateration method requires the conversion of RSSI to distance based on a path loss model. Since the simulator uses a path loss model, described in Equation (4), we assume that the conversion from RSSI to distance is based on perfect knowledge of the path loss parameters, and while it is not realistic to have a perfect representation of the path loss, the impact of imperfectly estimating the different parameters is out of the scope of this work. Furthermore, the conversion from RSSI to distance is negatively impacted by the small-scale fading and the unknown number of walls between the transmitter and receiver. As such, despite the perfect knowledge of path loss parameters, the localisation method will not be able to achieve a perfect localisation accuracy.

### 5.1. Simulation Setup

For all test results, the building plan used in the simulations is an exact replica of the building plan from Figure 3, which was used in the measurement campaign. This includes the locations of the CTUs which are placed on the wall facing the corridor, next to the doors, and while it would be possible to obtain greater localisation accuracy if the CTUs are placed in the centre of the rooms, the assumption of wall-mounted CTUs, as they also serve as HVAC control panels, does not allow for this. Instead, out of the four possible walls per room, preliminary results show that placing the CTUs on the front wall facing the corridor or the back wall provided the best performance. However, given the building layout where the back wall is likely filled with windows, and a control panel is most accessible by the door, placing the CTUs next to the doors is deemed to be the most appropriate choice.

The building plan is implemented in the simulation on a 10×10 cm^2^ grid. This means that each room has a fixed amount of locations where sensors can be placed. For the tests, the sensors are evenly spread across all possible grid locations in the simulator (covering all rooms). In particular, 10 sensor placements (with independent channel realisations) are considered in each possible location which totals 104,880 sensor placements, with 10,360 in each of the smaller rooms and 15,400 in each of the larger rooms.

### 5.2. Performance Metrics

The proposed methods are evaluated on their accuracy as a measure of the percentage of sensors whose location estimates are in the correct rooms. Since we are not interested in the precise location of the sensor, we do not evaluate the methods on their accuracy in terms of the distance between the estimated location and true location but on whether the room is detected correctly. Furthermore, the accuracy in each data point in the results is obtained from Monte Carlo based on the average accuracy over 100 individual tests where the CTU-to-CTU and sensor-to-CTU fingerprints each have a unique realisation of the spatial fading parameter ξ in the RSSI model.

### 5.3. Simulation Results

**Figure 5:**
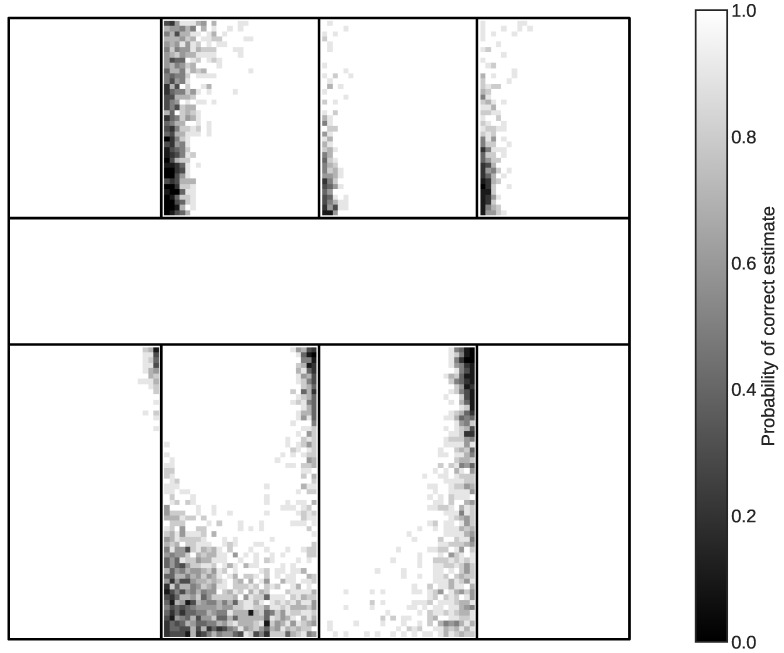
Heatmap showing the probability of correctly estimating a sensor using the single-sensor method for any location in the 8 rooms of the example building plan.

We first evaluate the proposed single-sensor method using the simulated environment from the measurement campaign. Figure 5 shows the computed probability of estimating correctly if a sensor is placed in a certain location in a room. It shows that while the method is largely able to estimate the correct room of the sensor, there are certain blind spots in the building where the MSE algorithm chooses the wrong room. The overall accuracy of the single-sensor method is 86.86%, which means that more than 1 in 10 randomly placed sensors will be estimated incorrectly. As a point of comparison, the extended fingerprinting and multilateration methods have an accuracy of 90.92% and 34.44% respectively. First, this shows that the multilateration, as expected, does not perform well in the indoor environment of the measurement campaign in spite of having perfect knowledge of the path loss parameters. A mixture of the noise introduced by the small-scale fading and the lack of knowledge on the number of walls between transmitter and receiver results in a localisation which estimated the correct room only 1 in 3 times. Second, we see that in spite of only using a single AP per room, the accuracy of the proposed automated localisation method is only 4.06 pp (percentage points) lower than the extensive fingerprinting method. The small difference between the automated and extensive fingerprinting methods shows that we do not lose significant performance when only using the CTUs as APs. Additionally, examples in the current SoA for room-level localisation [19,20] achieve a reported localisation accuracy of between 95 and 97%, and while this is greater than both the proposed single-sensor and extensive fingerprinting methods, the differences in propagation environment are unclear and the building layouts are not the same. As such, a closer comparison will be left for Section 5.5 where the performance of the proposed methods has been investigated for different propagation environments.

The heat map in Figure 5 indicates that each room has large parts with an accuracy of 100%. If the setup process of the BMS allows for the sensors to be placed in a known good location in the room during the localisation and then repositioned to their actual location after they have been linked to the CTUs, the correct room-mapping could be ensured. However, this process would introduce another manual step. As an alternative, we consider the possibility of increasing the accuracy using the multi-sensor methods. However, the accuracies of the multi-sensor localisation methods depend not only on the location of an individual sensor but also on the other sensors in the same group. Thus, the accuracy cannot be visualised in a heat map but will instead be evaluated along with the single-sensor method in the remaining results. For these results, the multilateration and extensive fingerprinting methods are not included. The multilateration is excluded due to its poor performance, which is deemed unacceptable for use in a BMS, and while the extensive fingerprinting does show a better performance than the automated version, it is reiterated that the automation requirements of the localisation methods for use in BMSs do not allow for the manual work required in the offline fingerprinting process. Instead, Section 5.5 summarises and compares the overall performance of the proposed multi-sensor methods with the extensive and SoA room-level fingerprinting methods.

The RSSI model in (4) is fitted specifically to a single environment and will therefore not be representative of all environments. As such, we next investigate the impact of changing the different parameters (path loss exponent α, WAF β, and noise variance $\sigma_{\xi }$) one at a time while keeping the remaining static. For each of the parameters, the values chosen are not representations of specific real environments. Instead, the values represent possible ranges that may be observed in different environments.

#### 5.3.1. Wall Attenuation Factor

**Figure 6:**
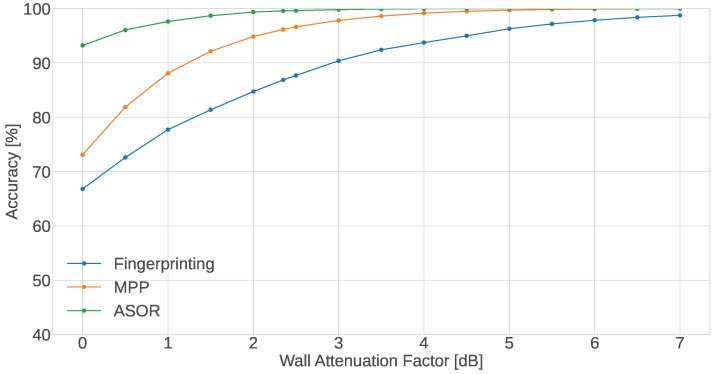
Accuracy of the proposed methods as a function of the WAF β.

Figure 6 shows the accuracy for WAF values ranging from 0 to 7 dB based on the range of attenuation values presented in [26]. It can be seen that the accuracy of all methods increases to around 100% for high values of WAF. This makes sense when we take into consideration the heat map of Figure 5. From the heat map, we can see that several of the blind spots are near the walls which the neighbouring CTUs are close to. This indicates that when the WAF is low, the closer distance to the neighbouring CTU along with the small-scale fading from the RSSI model has a bigger impact on the RSSI value than the attenuation caused by the wall. However, when the WAF is increased, the rooms are better separated, which increases the accuracy of the localisation. This is especially prevalent for the MPP method which at low WAF values only shows a slight increase in accuracy over the single-sensor method as compared to the accuracy offered by the ASOR method. This discrepancy between the ASOR and MPP methods seen for the lower WAF values is, however, expected. The MPP method has more ways it can fail where out of a group of 10 sensors it can incorrectly estimate the position of a few sensors while still estimating the remaining correctly. In these cases, the ASOR method would correct a few mistakes through the majority voting. However, it is important to note that the case for the MPP with multiple sensors is inherently different and arguably more difficult than when all sensors are in the same room. It is therefore difficult to directly compare the two methods. That being said, both multi-sensor methods show that for buildings with thick walls that offer a high attenuation, the localisation of sensors can likely be performed without errors.

The walls in the measurement environment are made from wood and plaster and are as such representative of the lower range of the signal attenuation. This means that for most environments, the walls will either provide a similar or more prominent attenuation. Translating this to the expected performance of the localisation methods, it means that the methods are expected to perform on par with or even better than the default environment results.

#### 5.3.2. Small-Scale Fading

**Figure 7:**
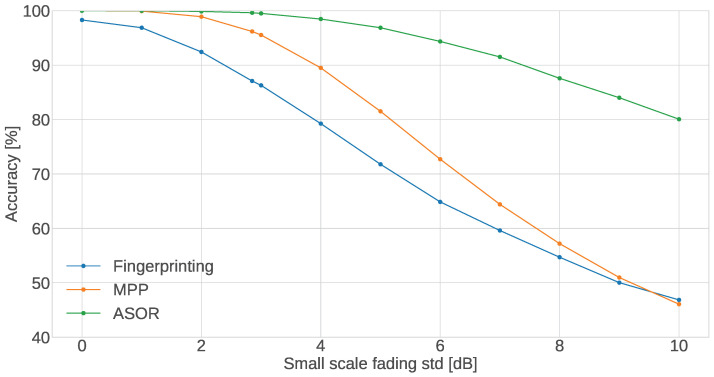
Accuracy of the proposed methods as a function of the std of the noise $\sigma_{\xi }$.

For the standard deviation (std) of the noise, the performance of the proposed methods, shown in Figure 7, also behaves as expected, where the accuracy of all methods decreases as the noise increases. However, even where there is no noise at all, the single-sensor method does not achieve 100% accuracy. This means that even without any distortion to the RSSI values, the attenuation caused by the walls of 2.35 dB is not high enough to overcome the difference in distance between the sensor and the two closest CTUs (the CTU in the same room as the sensor and the CTU in the adjacent room) when the sensor is up against the wall.

The ASOR method continues to show a general improvement in accuracy across all values of noise and even manages to keep an accuracy of 100% up to a std of 2 dB and staying above 99% up to 3 dB (which is above the measured value of 2.85 dB). Even at the high std values, the ASOR method manages to maintain an accuracy of over 80%, whereas both the single-sensor method and MPP method fall below 50%, and while the severe performance drop of the single-sensor method is expected, the similar performance of the MPP method further showcases the interesting consequence of the design of the method that was presented for the WAF results. Since the MPP method can only find solutions that conform to the specified amount of sensors in each room, there can be situations where more errors occur than the single-sensor method; if one sensor is estimated incorrectly, MPP must move an additional sensor to an incorrect room in order to conform to the sensor distribution. However, this case of worse performance seems to only happen when the conditions are very poor.

#### 5.3.3. Path Loss Exponent

**Figure 8:**
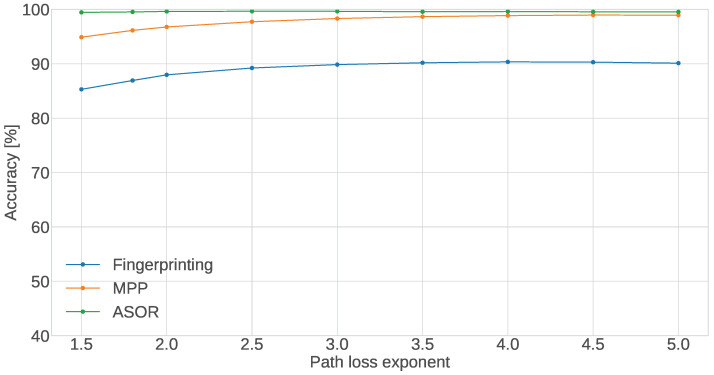
Accuracy of the proposed methods as a function of the path loss exponent α.

Lastly, the results for the path loss exponent, shown in Figure 8, show that the ASOR multi-sensor method is nearly invariant to changes in the path loss exponent while the accuracies of the MPP and single-sensor methods both increase slightly with the exponent. Since the path loss exponent is the factor that is most likely to change between buildings—even if the walls are made of the same material, changing the size of the rooms would impact the path loss exponent—being invariant to its changes is a desirable trait for localisation methods. It means that the same level of performance can be expected in most environments. The reason why the two methods are affected slightly by the exponent is due to the interaction in the RSSI model between the distance and wall attenuation. The logarithmic form of the model means that an increase in distance will affect the resulting RSSI value differently depending on the initial distance. This can be seen in Figure 4, where the model stagnates after some distance–at a small initial distance the change in distance results in a large change in RSSI and at a larger initial distance, the change in RSSI is small. By changing the path loss exponent we change when this stagnation point is, i.e., larger exponent values move the stagnation point to larger initial distances. What this means for the localisation is that when the exponent is small, there will be less separation between the RSSI values recorded at the different CTUs due to the particular room sizes in the building plan. This results in less meaningful information for the MSE calculations, which makes the methods more prone to failure. The reason why the ASOR method is not affected is likely due to the fact that the loss in information only affects a fraction of positions in each room where a sensor is more likely to be estimated in a neighbouring room. As such, the majority of sensors will still be estimated in the correct room and the few errors are corrected.

### 5.4. Real-World Validation

Since the previous results are all based on the simulator using the fitted RSSI model, we lastly perform a validation test where real-life measurements are used to perform localisation estimations with the proposed methods. The purpose of this test is to validate the simulation results by showing that the proposed methods work using measurements taken from real devices and by showing that the performance of the methods using these real-life measurements is similar to the performance of the simulated measurements. As such, the real-world validation will not provide additional exhaustive results showing the performance of the proposed methods given different sensor locations.

**Figure 9:**
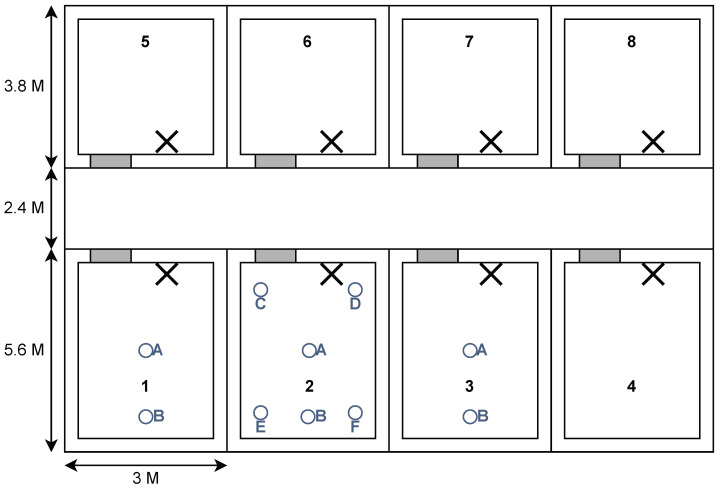
Overview of sensor locations for the real-world validation test. The building plan follows the same layout as the one used in the RSSI model fitting measurement campaign. The circles indicate the locations of sensors, and the letters and numbers are used as identifiers for each sensor (the number identifies which room the sensor is in, and the letters distinguish the position of a sensor in a room). The Xs indicate the locations of the CTUs in each room.

The measurements are taken separately from the measurements used in the RSSI model fitting but in the same environment as depicted in Figure 3. In total, we measure from 10 locations shown in Figure 9, following a similar measuring procedure as in the previous measurement campaign, excluding moving the transmitter to mitigate spatial fading. This includes the use of phones for both the role of sensors as well as CTUs. The specific locations are chosen based on a mixture of difficult and easy locations based on the heat map shown in Figure 5.

Since each localisation method requires slightly different information, the sensors are not estimated in the same way for each. The single-sensor method estimates each position individually and the MPP method estimates all 10 sensors as a single group knowing that there are 2 sensors in room 1, 6 sensors in room 2, and 2 sensors in room 3. However, the ASOR method has to divide the sensors into 3 groups, one for each room, and estimate each group individually. Since there are two groups with only 2 sensors, its accuracy in these has a risk of being worse than the single-sensor method. If the sensors in a group of 2 are estimated in two different rooms, the method will simply guess one of the rooms and estimate both sensors in that room. This is shown in Table 2, where we see that the ASOR method incorrectly estimates sensors 3A and 3B, one of which was incorrectly estimated by the single-sensor method. On the other hand, we see that the MPP method is able to correctly estimate all 10 sensors. It is able to overcome the problems imposed by sensor 3B by effectively utilising the information regarding the distribution of sensors. From Table 2, we additionally see that sensor 3B, which was incorrectly estimated, is estimated to be in room 7 instead of room 3. This error can be explained by considering that the room estimation is made based on the positions of the CTUs and that each CTU sensor link has a unique location-specific small-scale fading, whose standard deviation was measured to 2.85 dB. Compared to a wall attenuation of 2.3 dB and distances between different CTU sensor pairs being relatively similar, thus leading to comparable free-space path loss, it is not unreasonable that from time to time, cases will occur where specific combinations of location-specific fading lead to an incorrect room being the more likely position than the actually correct room.

Generating measurements using the simulator from the exact same locations, we obtain similar averaged accuracy results. Both the MPP and ASOR methods show a stable accuracy of 100 and 80% respectively, matching the real performance. Only the single-sensor method deviated slightly by dropping to an average accuracy of 83%. The main factor in this drop in accuracy is the locations, E, B, and F in room 2. As shown in Figure 5, these locations are significantly more prone to erroneous estimates, explaining the drop in accuracy for the single-sensor method. However, since there are more sensors in room 2, the ASOR method is still able to maintain a stable performance. With this in mind, we argue that the results shown in the previous tests are representative of the expected real-world performance.

### 5.5. Application of Proposed Methods to BMSs

Through the above results, we have shown that when there is a consistent amount of 10 sensors which are being estimated together using the proposed multi-sensor methods, the ASOR method performs better than both the single-sensor method as well as the MPP method. In the example environment, the ASOR method increases the localisation accuracy with 12.74 pp over the single-sensor method to an accuracy of 99.6% with the MPP method showing a 9.3 pp improvement reaching an accuracy of 96.16%, and while the improvement is not consistently large for all tested combinations of environment parameter values, the ASOR method does maintain its performance advantage. However, this does not mean that the ASOR method is unilaterally the best choice, and while ASOR does maintain a better performance across the board for the different environment parameters, this is only the case when there are enough sensors in each room to fully take advantage of the method. When there are too few sensors in each room, the ASOR method suffers greatly in terms of accuracy. In these cases, it would be beneficial to use the MPP method instead. As such, it will be up to the operator performing the installation of the sensors to decide which method is more appropriate given the specific situation. It is expected that a newly designed BMS for office space will support a wide variety of sensors and actuators, such as HVAC, indoor climate sensors, window actuators, light sensors and controllers, etc., and that many of these will be installed in each room. As such, it is likely that the ASOR method is able to perform with an accuracy close to what has been shown in this paper in most BMS configurations.

In spite of neither proposed method reaching 100% accuracy for all conceivable building environments, accuracies of 99.6% and 96.16% perform on par with or better than the current state of the art room level localisation which reaches between 95 and 97% accuracy [19,20]. However, as noted in Section 5.3, the propagation environments between the reported results for the SoA methods and the results presented for proposed methods in the default environment setting are not necessarily the same. When considering the presented performance of both the single- and multi-sensor methods, there are multiple cases of parameter combinations where all proposed methods outperform the reported performance of the SoA methods. Furthermore, the multi-sensor methods show a significant improvement to the extensive fingerprinting method for the same propagation environment. The results thereby show that the multi-sensor methods are effective methods of increasing the localisation accuracy of the proposed automated fingerprinting-based localisation method.

## 6. Conclusions

In this paper, we have presented two novel localisation methods with room-level accuracy which are based on an automated version of the RSSI fingerprinting approach. The proposed methods can be used for initialisation and maintenance of BMSs where the operator installing sensors and actuators is freed from excessive manual labour. The considered localisation methods use the fingerprinting principle to estimate the room in which one or more sensors are located. The reference fingerprints are RSSI measurements obtained by the CTUs deployed in each room. The considered baseline fingerprinting method that estimates the most likely room for each sensor individually, on average, maps 1 out of 8 sensors incorrectly. The proposed methods that take advantage of knowledge present in the building plan regarding the distribution of sensors in each room reduce the error rate to 1 out of 20 sensors (if the number of sensors per room is known) or as few as 1 per 200 sensors (if a group of sensors are deployed and detected together for one room at a time).

## Figures and Tables

**Table 1:** Fitted parameters of Equation (4).

$P_{0}$	α	β	$\sigma_{\xi }$
47.75 dBm	1.8	2.3 dB	2.85 dB

**Table 2:** Sensor estimates following the positions in Figure 9 for the different localisation algorithms (SS denotes the single-sensor method). In the table, each entry denotes the room in which the sensor was estimated to be.

	1A	1B	2A	2B	2C	2D	2E	2F	3A	3B	Accuracy
SS	1	1	2	2	2	2	2	2	3	7	9/10
MPP	1	1	2	2	2	2	2	2	3	3	10/10
ASOR	1	1	2	2	2	2	2	2	7	7	8/10

## Data Availability

Data are contained within the article.
